# *C9orf72* ALS‐causing mutations lead to mislocalization and aggregation of nucleoporin Nup107 into stress granules

**DOI:** 10.1002/1873-3468.70156

**Published:** 2025-09-01

**Authors:** Saygın Bilican, Yara Nabawi, William Hongyu Zhang, Dunja Petrovic, Markus Wehrmann, Sara Muñoz‐García, Seda Koyuncu, David Vilchez

**Affiliations:** ^1^ Institute for Integrated Stress Response Signaling, Faculty of Medicine University Hospital Cologne Germany; ^2^ Cologne Excellence Cluster for Cellular Stress Responses in Aging‐Associated Diseases (CECAD) University of Cologne Germany; ^3^ Institute for Genetics University of Cologne Germany; ^4^ Center for Molecular Medicine Cologne (CMMC) University of Cologne Germany

**Keywords:** Amyotrophic lateral sclerosis, *C. elegans*, iPSC‐disease modeling, Nucleoporins, Proteostasis, Stress granules

## Abstract

Amyotrophic lateral sclerosis (ALS) is a fatal disorder caused by motor neuron degeneration. Hexanucleotide repeat expansions in the *C9orf72* gene, the most common genetic cause of ALS (C9‐ALS), drive toxicity through different mechanisms. These pathological changes include alterations in stress granules (SGs), ribonucleoprotein complexes formed under stress conditions. Here, we show that G3BP1, a core component of SGs, exhibits enhanced interaction with the nucleoporin Nup107 in motor neurons derived from patient iPSCs carrying *C9orf72* mutations. Moreover, Nup107 colocalizes with SGs and aggregates in C9‐ALS motor neurons. Notably, knockdown of *npp‐5*, the *Caenorhabditis elegans* ortholog of *Nup107,* alleviates ALS‐associated phenotypes in worm models, including reduced lifespan and impaired motility. Together, our findings provide insights into disease‐related changes in C9‐ALS pathogenesis.

## Graphical abstract

Mutations in the *C9orf72* gene represent the most common genetic cause of amyotrophic lateral sclerosis (ALS), a fatal neurodegenerative disease. Using patient‐derived neurons and *C. elegans* models, we find that the nucleoporin Nup107 is dysregulated in *C9orf72*‐associated ALS. Conversely, reducing Nup107 levels mitigates disease‐related changes. These findings provide insights into the molecular mechanisms underlying ALS.

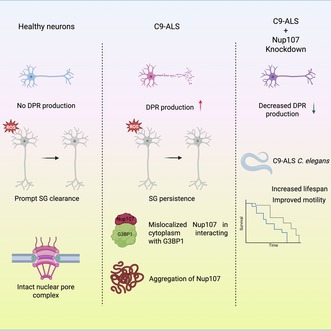

## Abbreviations

**ALS**, amyotrophic lateral sclerosis

**C9‐ALS**, *C9orf72*‐amyotrophic lateral sclerosis

**DPR**, dipeptide repeats

**EMSA**, electrophoretic mobility shift assay

**HRE**, hexanucleotide repeat expansion

**iMN**, iPSC‐derived motor neuron

**iPSC**, induced pluripotent stem cell

**NCT**, nucleocytoplasmic transport

**NPC**, nuclear pore complex

**RAN**, repeat‐associated non‐AUG translation

**RBP**, RNA‐binding proteins

**SG**, stress granule

Amyotrophic lateral sclerosis (ALS) is the most common motor neuron disease, affecting approximately 6 in 100 000 people [1, 2]. ALS leads to the degeneration of upper and lower motor neurons, resulting in muscle weakness, atrophy, and ultimately, death [3]. While the majority of ALS cases are sporadic, around 10% are familial and linked to mutations in one of over 30 different genes [3, 4, 5]. An intronic GGGGCC (*G*_*4*_ *C*_*2*_) hexanucleotide repeat expansion (HRE) in the first intron of the *C9orf72* gene is the most common mutation causing ALS (C9‐ALS), accounting for up to 40% of familial and 10% of sporadic cases [6, 7, 8, 9].

The pathogenic mechanisms of C9‐ALS are complex and nonmutually exclusive. The HRE mutation reduces *C9orf72* mRNA and protein levels, leading to a loss of function [10, 11, 12]. Additionally, GC‐rich DNA and RNA repeats form G‐quadruplexes that sequester nucleic acid‐binding proteins, disrupting transcription and generating RNA foci [13, 14, 15]. The mutant transcript can also undergo repeat‐associated non‐AUG (RAN) translation, producing dipeptide repeats (DPRs) [16, 17, 18]. These DPRs exert proteotoxic effects through their aggregation and binding to low‐complexity‐domain containing proteins [16, 19, 20, 21, 22, 23, 24, 25, 26, 27, 28].

The toxic effects of RNA foci and DPRs in C9‐ALS are closely linked to cellular stress responses, particularly stress granules (SGs) [29, 30]. SGs are membrane‐less organelles that form in response to different internal and external stressors but rapidly disassemble upon stress removal [31, 32]. Their main components include mRNA, RNA‐binding proteins (RBPs), 40S ribosomal subunits, and translation initiation factors [33, 34, 35]. One of the core proteins of SGs is Ras GTPase‐activating protein‐binding protein 1 (G3BP1) [36, 37]. G3BP1, along with other core SG proteins such as TIA1, TTP, and FMRP, facilitates SG formation by recruiting mRNA and ribosomal subunits [38, 39]. In turn, this process attracts other proteins containing low‐complexity and intrinsically disordered domains, leading to the formation of a dynamic, liquid‐like shell through liquid–liquid phase separation [40, 41]. SGs are highly dynamic structures, with the mobility of their components influenced by ATP levels, which regulate their movement, fusion, and fission within the cell [42, 43].

However, the low‐complexity and intrinsically disordered domains of SG components are also prone to aggregation, a process that can lead to aberrant and persistent granules [41]. Along these lines, dysregulated SG dynamics (e.g., excessive formation, aggregation, impaired disassembly, and persistence) are a hallmark of ALS [41, 44, 45, 46]. ALS‐relevant proteins such as TDP‐43, FUS, and DPRs, as well as HRE‐RNA, accumulate into SGs, suggesting that SG alterations may contribute to ALS pathophysiology [23, 47, 48, 49].

In addition to affecting SG dynamics, ALS‐causing *C9orf72* mutations also disrupt nucleocytoplasmic transport (NCT) pathways [50, 51, 52, 53]. The *C9orf72* HRE can be transcribed in both sense and antisense directions, producing RNA repeats that bind to RanGAP1, a key regulator of NCT, thereby impairing nuclear import [53]. In *Drosophila melanogaster*, expression of expanded *G*_*4*_ *C*_*2*_ repeats induces cellular toxicity, resulting in a rough eye phenotype [50, 53]. An unbiased genetic screen in fruit flies identified 18 components of the NCT pathway as genetic modifiers of C9‐ALS‐related toxicity. Among them, loss of the nuclear pore complex (NPC) components Nup50 and Nup153 exacerbates the rough eye phenotype, whereas loss of Nup160 and Nup107 suppresses it [50]. Another study in fruit flies demonstrated that expanded *G*_*4*_ *C*_*2*_ repeats reduce the levels of several NPC components, including Nup50, Nup98, and Nup214 [54]. A similar reduction in nucleoporins, such as Nup50, Nup98, Nup133, and Nup107, was observed in aged human iPSC‐derived neurons expressing different ALS‐related *C9orf72* mutant variants [55]. Importantly, various NPC components, such as Nup50, Nup62, Nup88, Nup205, and Nup358, have been reported to mislocalize into SGs under oxidative or hyperosmotic stress in naïve human cell lines [56]. Among these, Nup62 exhibits nuclear depletion and cytoplasmic mislocalization into ribonucleoprotein granules in C9‐ALS iPSC‐derived neurons, leading to TDP‐43 accumulation in the cytoplasm [57].

In this study, we demonstrate that the SG core component G3BP1 gains an interaction with the NPC subunit Nup107 in iPSC‐derived motor neurons (iMNs) from ALS patients carrying *C9orf72* mutations. We also find that Nup107 is mislocalized in C9‐ALS cells, forming cytoplasmic foci. These cells display increased SG assembly and slower disassembly rates, with Nup107 localizing to the granules. Moreover, Nup107 aggregation is exacerbated under oxidative stress in C9‐ALS cells. Notably, knockdown of the *Caenorhabditis elegans* ortholog of *Nup107* in a C9‐ALS model mitigates disease‐associated phenotypes, including shortened lifespan, reduced motility, and DPR accumulation. Together, our findings reveal a link between C9‐ALS and Nup107 dysregulation, with potential pathological implications.

## Materials and methods

### iPSC lines and culturing

The C9‐1 (CS29iALS‐C9n1; HRE: ~6000–8000 bp repeat expansion, RRID:CVCL_W559) and C9‐2 (CS30iALS‐C9n1; HRE: ~2400 bp repeat expansion, RRID:CVCL_W560) iPSC lines were obtained from the Cedars‐Sinai RMI iPSC Core. The isogenic control line for C9‐1 (CS29iALS‐C9n1.ISOT2RB4) in which the *C9orf72* mutation was corrected using CRISPR‐Cas9, was generated and provided by the Cedars‐Sinai RMI iPSC Core. Control (SAPi001‐A, RRID:CVCL_C0NK) and FUS^P525L^ (SAPi001‐A‐1) iPSC lines were generously provided by I. Bozzoni and A. Rosa [58]. TDP‐43^M337V^ iPSCs (CiRA00024, RRID:CVCL_T781) were obtained from RIKEN Bioresource Research Center [59].

iPSCs were maintained in mTeSR1 medium (#85850; Stem Cell Technologies, Cologne, Germany), and cultured on tissue culture‐treated plates coated with Geltrex (#A1413202; Thermo Fisher Scientific, Dreieich, Germany) at 37 °C in a humidified incubator with 5% CO_2_. For passaging, cells were detached using Accutase (#A1110501; Thermo Fisher Scientific). Briefly, the medium was removed, and cells were incubated with Accutase for 3–5 min. Accutase was then neutralized with mTeSR1, and the cell suspension was centrifuged at 300 ***g*** for 5 min. The cells were then reseeded onto Geltrex‐coated plates at the desired density in mTeSR1 supplemented with 10 μm ROCK inhibitor (# ab120129; Abcam, Cambridge, UK) for the first day of culture.

All cell lines were tested for mycoplasma contamination at least once every 3–6 months, and no contamination was detected. Over the past 3 years, the control iPSC line (SAPi001‐A) and the FUS^P525L^ iPSC line (SAPi001‐A‐1) were authenticated in our laboratory by short tandem repeat (STR) profiling using the following microsatellite markers: D17S1303, D16S539, vWA, THO1, CSF1PO, and TPOX, as described in Ref. [60]. During the same period, the other iPSC lines used in this study were directly thawed from early stocks of the original vials provided by the suppliers, who performed STR‐based authentication as documented in each line's Certificate of Analysis.

### Differentiation of iPSCs into motor neurons

The differentiation protocol was adapted from Hill *et al*. [61]. Briefly, iPSCs were cultured until they reached 80–90% confluency. The first day of media replacement was defined as Day 0 (d0). From d0 to d6, mTeSR1 was replaced with Differentiation Media, consisting of a 1 : 1 mixture of DMEM F‐12 (#11320074; Thermo Fisher Scientific) and Neurobasal Media (#21103049; Thermo Fisher Scientific) supplemented with 1× B27 (#12587‐010; Thermo Fisher Scientific), 1× N2 (#175020‐01; Thermo Fisher Scientific), 1× non‐essential amino acids (#11140050; Thermo Fisher Scientific), 100 U·mL^−1^ penicillin/streptomycin (#15070063; Thermo Fisher Scientific), 1× GlutaMAX (#35050061; Thermo Fisher Scientific). Additionally, the medium was supplemented with 1 μm smoothened agonist (SAG) (#566661; Sigma, Schnelldorf, Germany), 1 μm retinoic acid (RA) (#R2625; Sigma), 10 μm SB‐431542 (#130‐106‐275; Miltenyi Biotec, Bergisch Gladbach, Germany), and 0.1 μm LDN‐193189 (#130‐103‐925; Miltenyi Biotec). From d7 to d13, the Differentiation Media was supplemented with 1 μm SAG, 1 μm RA, 4 μm SU‐5402 (#SML0443‐5MG; Sigma), and 5 μm DAPT (#D5942; Sigma) instead.

On d14, the cells were passaged using Accutase and plated onto laminin‐coated tissue culture plates. Briefly, plates were first coated with 1.5 μg·mL^−1^ poly‐L‐ornithine (L‐PO) (# P3655; Sigma) in PBS and incubated overnight at 4 °C. The following day, L‐PO was washed off twice with PBS and once with DMEM/F‐12. Plates were then coated with 10 μg·mL^−1^ mouse laminin (#23017‐015; Thermo Fisher Scientific) in DMEM/F‐12 and incubated overnight at 4 °C. Cells were subsequently seeded and maintained in Neuron Media (Neurobasal medium with 1× B27, 1× N2, 1× non‐essential amino acids, 100 U penicillin/streptomycin, 1× GlutaMAX, 10 ng·mL^−1^ BDNF [#450‐02; Peprotech, Hamburg, Germany], and 10·ng mL^−1^ GDNF [#450‐10; Peprotech]). Cells were allowed to mature for a minimum of 2 days before proceeding with subsequent experiments.

### G3BP1‐GFP reporter iPSC lines

G3BP1‐GFP reporter control iPSCs were previously generated in our laboratory via CRISPR‐Cas9–mediated knock‐in, as described in Ref. [47]. Using the same protocol, we generated the G3BP1‐GFP C9‐2 iPSC line in this study. The guide RNA (gRNA) was prepared by annealing 0.02 nm crRNA (Table S1) and tracrRNA (#1072532; IDT Alt‐R, Coralville, IA, USA) at 95 °C for 5 min, followed by cooling and storage at −20 °C. For transfection, the gRNA, Cas9 protein (1 μm), and repair template (2 μg) were mixed with FuGENE® HD and Opti‐MEM™ and incubated prior to addition to cells. copGFP‐positive cells were isolated by fluorescence microscopy and serial dilution. Monoclonal colonies were expanded, and successful G3BP1‐GFP knock‐in was confirmed by sequencing and western blot analysis.

### Generation of lentiviral particles

The Tet‐pLKO‐puro plasmid was a gift from D. Wiederschain (#21915; Addgene, Watertown, MA, USA) [62]. NUP107‐targeting shRNA primer sequences were obtained from The RNAi Consortium shRNA Library [63] and synthesized by Integrated DNA Technologies (IDT). Primer sequences and associated sequencing primers are listed in Table S1. The protocol for generating lentiviral plasmids, and particles was adapted from Ref. [64] and the RNAi Core Facility of Academia Sinica (Taipei, Taiwan).

For annealing, 9 μL of each 100 μm primer was mixed with 2 μL of 10× Primer Annealing Buffer (1 m potassium acetate, 0.3 m HEPES‐KOH pH 7.4, 20 mm magnesium acetate). Annealing was performed by sequential incubation for 5 min at the following temperatures: 95 °C, 78 °C, 74 °C, 70 °C, 67 °C, 63 °C, 60 °C, 56 °C, 53 °C, 50 °C, 48 °C, 46 °C, 44 °C, 42 °C, 40 °C, and 39 °C. This was followed by six cycles at 37 °C (5 min each), decreasing the temperature by 1 °C per cycle, and five cycles at 30 °C (10 min each), with 2 °C temperature decrements per cycle. The Tet‐pLKO‐puro plasmid was digested with *AgeI‐HF* (R3552S; NEB, Frankfurt am Main, Germany) and *EcoRI‐HF* (R3101S; NEB). Ligation was performed with 50 ng of digested vector and 1 μL annealed primers using T4 DNA Ligase (M0202S; NEB) at 16 °C overnight. Ligation products were transformed into *E. coli* DH10α and selected using ampicillin. Positive colonies were verified by sequencing (Table S1).

Lentiviral particles were produced using psPAX2 (#12260; Addgene) and pMD2.G (#12259; Addgene), gifts from D. Trono, following the protocol adapted from REFs [64, 65, 66]. HEK293FT cells were plated in 10‐cm dishes and transfected at approximately 70–80% confluency with 30 μL FuGENE® HD (E2692; Promega, Walldorf, Germany), 5 μg psPAX2, 5 μg pMD2.G, and 10 μg lentiviral plasmid in 600 μL Opti‐MEM™ (Thermo Fisher Scientific; 31985070). After 15‐min incubation at room temperature, the mix was added to cells. The next day, the medium was replaced with fresh antibiotic‐free HEK293 medium. Viral supernatants were collected on Days 2, 3, and 4 post transfection and stored at 4 °C.

To concentrate lentiviral particles, pooled supernatants were centrifuged at 800 ***g*** for 10 min to remove debris, mixed with 10 mL of 4× lentiviral concentrator solution (400 g/L PEG‐8000, 70 g/L NaCl in 1× PBS), and incubated overnight at 4 °C. The next day, samples were centrifuged at 1600 ***g*** for 1 h at 4 °C, and the viral pellet was resuspended in DMEM/F‐12 (11320074; Thermo Fisher Scientific) and stored at −80 °C.

### Transduction of iPSCs with lentiviral particles

iPSCs at 40–50% confluency were transduced with 7.2 mL of transduction mix consisting of 6.6 mL mTeSR1 (Stem Cell Technologies), 600 μL concentrated lentiviral particles, and 10 μg·mL^−1^ polybrene (H9268‐5G; Sigma). A second transduction with freshly prepared mix was performed 1 h later. Three days post transduction, 1 μg·mL^−1^ puromycin (A1113803; Thermo Fisher Scientific) was added to the culture medium for selection and maintained throughout routine culture, except during the 4 days preceding experiments, when cells were cultured in puromycin‐free medium. To induce shRNA expression, cells were treated with 2 μg·mL^−1^ doxycycline (HY‐N0565B; MedChemExpress, Sollentuna, Sweden) for 2 days, followed by 1 day in doxycycline‐free medium prior to analysis.

### SG induction and disassembly

SG assembly was induced by treating the cells with 500 μm sodium arsenite (#106277; Sigma) For recovery experiments, cells were washed once with DPBS (#14200075; Thermo Fisher Scientific) after the treatment and maintained in fresh culture medium. The cells were then fixed with 4% paraformaldehyde (PFA) for 15 min and stained for immunofluorescence microscopy according to the following protocol. SGs were manually counted using ImageJ [67].

### Immunofluorescence staining

Cells were plated onto glass coverslips and fixed with 4% PFA (#04018‐1; Polysciences, Hirschberg an der Bergstrasse, Germany) for 15 min at room temperature, followed by two washes with 1× PBS. Cells were then permeabilized using PBS containing 0.2% (v/v) Triton X‐100 and blocked with 3% bovine serum albumin (BSA) in PBS. Cells were incubated with primary antibodies diluted in 3% BSA in PBS, including mouse anti‐G3BP1 (#ab56574, 1 : 300; Abcam), rabbit anti‐G3BP1 (#MBL‐RN048PW, 1 : 500; Biozol, Hamburg, Germany), rabbit anti‐TIA1 (#12133‐2‐AP, 1 : 500; Proteintech, Planegg‐Martinsried, Germany), rabbit anti‐NUP107 (#19217‐1‐AP, 1 : 50 for iPSCs and 1 : 250 for iMNs; Proteintech), rabbit anti‐DDX6 (#14632‐1‐AP, 1 : 200; Proteintech), and goat anti‐Choline Acetyltransferase (#AB144P, 1 : 100; Merck, Darmstadt, Germany). Primary antibody incubation was carried out for 1.5 h at room temperature or overnight at 4 °C in a humidified chamber.

Following primary antibody incubation, coverslips were washed three times with 1× PBS and subsequently incubated for 45 min with secondary antibodies and 10 μg·mL^−1^ Hoechst 33342 (#H3570; Thermo Fischer Scientific, Dreieich, Germany), all diluted in 3% BSA in PBS. The secondary antibodies used were Goat anti‐Mouse IgG (H + L) Highly Cross‐Adsorbed Secondary Antibody, Alexa Fluor 488 (#A‐11029, 1 : 300; Thermo Fisher Scientific), Goat anti‐Rabbit IgG (H + L) Cross‐Adsorbed Secondary Antibody, Alexa Fluor 594 (#A‐11012, 1 : 300; Thermo Fisher Scientific), Donkey anti‐Goat IgG (H + L) Cross‐Adsorbed Secondary Antibody, Alexa Fluor 488 (#A‐11055, 1 : 500; Thermo Fisher Scientific), Donkey anti‐Rabbit IgG (H + L) Highly Cross‐Adsorbed Secondary Antibody, Alexa Fluor 555 (#A‐31572, 1 : 500; Thermo Fisher Scientific), and Donkey anti‐Mouse IgG (H + L) Highly Cross‐Adsorbed Secondary Antibody, Alexa Fluor 647 (#A‐31571, 1 : 500; Thermo Fisher Scientific). Unbound secondary antibodies were removed by three washes with PBS, followed by a final rinse with ddH_2_O. Coverslips were dehydrated using 100% ethanol and left to dry in the dark. Once dried, coverslips were mounted onto microscope slides using ProLong Diamond Antifade Mountant (#P36961; Thermo Fisher Scientific) or FluorSave Reagent (#345789; Merck). Imaging was performed using a Zeiss Axio Imager Z.1 microscope.

### Quantification of SG/Nup107 colocalization and encapsulation

SG colocalization and encapsulation analysis was performed using the Zeiss ZEN image analysis software (version 3.12). The localization of SG and NUP107 condensates was verified by measuring the overlap of cross‐sectional signal intensity using the in‐built profile analysis tool and visual assessment. Peaks with overlapping signal maxima corresponded to colocalization, while NUP107 peaks at a local SG signal minima that are also directly adjacent to SG peaks corresponded to the encapsulation of NUP107 by SGs.

Motor neurons were quantified individually, while iPSC images were analyzed by dividing each image into four quadrants, determining an average value per cell per quadrant, and using this value for the statistical analysis, with each image yielding four values.

### Western blotting

Protein extraction was performed using either RIPA buffer (50 mm Tris‐Cl pH 7.5, 150 mm NaCl, 1% Triton X‐100, 1% sodium deoxycholate, 0.1% SDS, 1 mm EDTA) or a nondenaturing native lysis buffer (150 mm NaCl, 50 mm HEPES pH 7.4, 1 mm EDTA and 1% Triton X‐100). Both buffers were supplemented with cOmplete Mini Protease Inhibitor Cocktail (#11836153001; Roche, Penzberg, Germany) and 1 mm PMSF. Protein concentrations were determined using the BCA assay (A55865; Thermo Fisher Scientific). Equal amounts of protein were separated via SDS/PAGE and transferred onto polyvinylidene difluoride (PVDF) membranes (Millipore, Darmstadt, Germany). Membranes were blocked with 3% BSA in TBS‐T for 1 h at room temperature, followed by overnight incubation at 4 °C with primary antibodies diluted in blocking solution (mouse anti‐poly‐GA [#MABN889, 1 : 1000; Merck], mouse anti‐α‐tubulin [#T6199, 1 : 5000; Sigma], and rabbit anti‐NUP107 [#19217‐1‐AP, 1 : 5000; Proteintech]). Immunoblotting for cell death analysis was performed using antibodies against Phospho‐RIP (Ser166) (#65746, clone D1L3S, 1 : 1000; Cell Signaling, Frankfurt am Main, Germany), RIP (#3493, clone D94C12, 1 : 1000; Cell Signaling), Cleaved Caspase‐3 (#9661, 1 : 2000; Cell Signaling), and anti‐β‐actin (#8226, 1 : 5000; Abcam).

The next day, membranes were washed three times with TBS‐T (5 min per wash) and incubated for 1 h at room temperature with secondary antibodies diluted in blocking solution (Donkey HRP AP anti‐Mouse IgG (H + L) [#715‐035‐1500, 1 : 10 000; Jackson Immuno Research, West Grove, PA, USA], Donkey HRP AP anti‐Rabbit IgG (H + L) [Jackson Immuno Research, #715–035‐1520, 1:10 000]). Following secondary antibody incubation, membranes were washed three times with TBS‐T (5 min per wash) and developed using Immobilon Western Chemiluminescent HRP Substrate (#WBKLS0500; Merck). The signal was detected using the FUSION SOLO S imaging system (Vilber).

### Filter trap assays

Proteins were extracted using denaturing native lysis buffer (150 mm NaCl, 50 mm HEPES pH 7.4, 1 mm EDTA, and 1% Triton X‐100), and lysates were sonicated for 30 s at 40% amplitude using the Bandelin Electronic Sonopuls Ultrasonic Homogenizer Mini20. Cell debris was removed by centrifugation at 3000 ***g*** for 4 min at 4 °C, and the supernatant was transferred to a new tube. Protein concentrations were determined using the BCA assay (A55865; Thermo Fisher Scientific). To assess NUP107 and α‐tubulin (loading control) levels by western blot, equal amounts of total protein were separated by SDS/PAGE, transferred to PVDF membranes (Millipore) and immunoblotted as described above.

To assess aggregated Nup107 levels by filter trap, equal amounts of total protein from each sample were adjusted to 100 μL and supplemented with a final concentration of 0.5% SDS. The slot blot apparatus (#1706542; BIO‐RAD, Feldkirchen, Germany) was assembled with a cellulose acetate membrane (#516‐5020; VWR, Darmstadt, Germany) and equilibrated with native buffer containing 0.5% SDS. Samples were loaded into the slots and allowed to pass through the membrane completely. The membrane was then washed with 0.2% SDS in ddH_2_O. The membrane was then blocked with 3% BSA in TBS‐T for 1 h and incubated overnight at 4 °C with the primary Nup107 antibody (rabbit anti‐NUP107 [#19217‐1‐AP, 1 : 5000; Proteintech]). The next day, the membrane was washed three times with TBS‐T and incubated with the secondary antibody IRDye 800CW Donkey anti‐Rabbit IgG (H + L) (#926‐32 213, 1 : 10 000; LI‐COR, Bad Homburg vor der Höhe, Germany) for 1 h. After incubation, the membrane was washed again with TBS‐T, and imaging was performed using the Odyssey M Imaging System.

### Soluble and insoluble protein fractionation

Soluble and insoluble protein fractionation was performed as previously described [68]. Briefly, cells were lysed in ice‐cold 1% Triton X‐100 in phosphate‐buffered saline (PBS) supplemented with 2 mM sodium orthovanadate and an EDTA‐free protease inhibitor cocktail (Roche) on ice. Lysates were homogenized using a 27‐gauge syringe needle. Protein concentrations were then determined using a standard BCA protein assay (A55865; Thermo Scientific).

Equal protein amounts of starting lysates were centrifuged at 15000 *g* for 30 min at 4 °C. The supernatant, representing the 1% Triton X‐100–soluble fraction, was collected. The resulting pellets were washed four times with ice‐cold 1% Triton X‐100 in PBS, resuspended in 1% SDS, and incubated for 1 h at 60 °C. Triton X‐100–insoluble fractions were then collected by centrifugation at 15 000 ***g*** for min at 4 °C. Both soluble and insoluble fractions were subsequently used for western blot analysis with rabbit anti‐NUP107 (#19217‐1‐AP, 1 : 5000; Proteintech) and mouse anti‐α‐tubulin (#T6199, 1 : 5000; Sigma).

### Co‐immunoprecipitation and proteomics sample preparation

Proteins were extracted from neuronal cultures using SDS‐free RIPA buffer and homogenized with a 27‐gauge needle. After homogenization, the samples were centrifuged at maximum speed for 10 min at 4 °C, and the resulting supernatants were collected. For immunoprecipitation, 300 μg of protein lysates were incubated with 2 μg of monoclonal mouse anti‐G3BP1 antibodies (#ab56574; Abcam) on ice for 1 h. Next, 50 μL of Protein A microbeads (#130‐071‐001; Miltenyi Biotec) were added, and the mixture was incubated at 4 °C on a rotator for 2 h. All subsequent steps were performed in a cold room until the digestion step.

μColumns (130‐042‐701; Miltenyi Biotec) placed on magnetic holders were pretreated with 200 μL of SDS‐free RIPA buffer. The protein‐antibody‐bead complex was loaded onto the columns and allowed to pass through by gravity flow. The columns were washed three times with 200 μL of Wash Buffer I (50 mm Tris‐Cl pH 7.5, 150 mm NaCl, 0.05% Triton X‐100, 5% glycerol), followed by five washes with Wash Buffer II (50 mm Tris‐Cl pH 7.5, 150 mm NaCl).

Bound proteins were digested directly on the columns with 25 μL digestion buffer (2 m Urea, 7.5 mm ammonium bicarbonate, 1 mm DTT, 5 ng·mL^−1^ trypsin) at room temperature for 30 min, then eluted using 50 μL of elution buffer (2 m Urea, 7.5 mm ammonium bicarbonate, 5 mm CAA). Digestion was completed overnight in the dark. The next day, formic acid was added to a final concentration of 4%, and samples were centrifuged at maximum speed for 5 min. The supernatants were loaded onto SDP‐RP StageTips and sent for label‐free quantitative proteomics at the CECAD Proteomics Core Facility. Raw proteomics data were analyzed using Perseus v1.6.13.0 [69].

### *C. elegans* strains and maintenance

*C. elegans* were maintained under standard conditions at 20 °C on nematode growth medium (NGM) plates seeded with the OP50 *E. coli*. The KRA315 (*snb‐1p*::*C9* ^*ubi*^ + *myo‐2p*::GFP) and KRA317 (*snb‐1p*::*ΔC9* ^*ubi*^ + *myo‐2p*::GFP) strains were kindly provided by Paschalis Kratsios [70]. For RNAi experiments, late‐L4 stage *C. elegans* were fed with the *E. coli* HTT115 strain carrying either the L4440 empty vector control or L4440 expressing double‐stranded RNA targeting *npp‐5*. The *npp‐5* and *npp‐10* RNAi constructs were obtained from the Ahringer library and were sequence‐verified prior to use (Table S1).

### *C. elegans* lifespan assays

Synchronization of *C. elegans* was carried out using a bleaching protocol. Adult hermaphrodites containing eggs were treated with a bleaching solution (1.5% v/v NaClO, 0.75 m KOH in ddH_2_O) until the eggs were released and the adults were dissolved. The eggs were then incubated overnight in M9 buffer to allow hatching and synchronization at the L1 larval stage. L1 larvae were transferred to OP50‐seeded plates and grown at 20 °C until they reached the first day of adulthood.

For lifespan assays, 96 worms per condition were transferred onto RNAi plates and monitored either daily or every other day [71]. Worms exhibiting a protruding vulva, bagging phenotype, or that were lost during the experiment were censored. Lifespan data were analyzed using GraphPad Prism (version 9.3) and statistical significance was calculated by the log‐rank (Mantel‐Cox) test. OASIS software was used to determine mean lifespan. *P*‐values were calculated for comparisons between two groups within a single experiment.

### *C. elegans* motility assays

Day‐5 adult *C. elegans* were placed in 20 μL of M9 buffer and allowed to acclimate for 30 s. Body bends were defined as changes in body direction. The number of body bends was counted over the next 30 s [72].

### Quantitative real‐time PCR (qRT‐PCR)

Total RNA was extracted using RNAbee (Tel‐Test) from approximately 2000 adult day 5 *C. elegans* subjected to RNAi treatment. cDNA was synthesized from 1 μg of total RNA using the qScript Flex cDNA Synthesis Kit (Quantabio, Beverly, MA, USA). SYBR Green–based real‐time qPCR was performed using a 1:20 dilution of cDNA on the CFX384 Real‐Time System (Bio‐Rad). Gene expression levels were quantified using the comparative $2^{\Delta \Delta C_{t}}$ method, with the geometric mean of *cdc‐42*, *pmp‐3*, and *Y45F10D.4* used as housekeeping genes [73]. Primer sequences used for this assay are listed in Table S1.

### Bacterial expression and purification of G3BP1 and Nup107

The protocol for protein expression and purification was adapted from Llamas et al. 2023 [74]. Briefly, human G3BP1 and NUP107 cDNA were cloned into the pGEX‐6P‐1 vector, which contains a 6x His‐Tag, using GeneArt Gibson Assembly HiFi Master Mix (#A46627; ThermoFischer) and the primers listed in Table S1. After confirming the constructs by sequencing, *E. coli* BL21(DE3) was transformed with the vector containing the respective cDNA. The bacteria were cultured at 37 °C for initial growth, and the culture was further incubated at 18 °C overnight after the addition of 0.25 mm isopropyl 1‐thio‐β‐d‐galactopyranoside to induce protein expression.

The bacterial culture was centrifuged at 25 000 ***g*** at 4 °C for 1 h and then sonicated. The lysates were clarified by centrifugation at 15 000 ***g*** at 4 °C for 1 h. His‐Tag‐containing proteins were purified using HisPurCobalt Resin (#89964; Thermo Fisher Scientific) via affinity chromatography. The His‐tag was removed by treatment with TEV protease during overnight dialysis. The dialyzed proteins were subjected to a second affinity chromatography step to remove contaminants, and enriched fractions were concentrated using Amicon Ultra‐15 filters (#10403892; Merck). Protein concentration was determined with NanoDrop 8000. Single‐use aliquots of the purified proteins were snap‐frozen in liquid nitrogen and stored at −80 °C. Each protein fraction obtained during the purification process was analyzed by SDS/PAGE.

### Electrophoretic mobility shift assay (EMSA)

The EMSA protocol was adapted from Hsieh et al (2016) and Celona et al (2017) [75, 76]. The (GGGGCC)_6.5_ and (AAAACC)_6.5_ probes, tagged with TYE 563 at their 5'end, were obtained from IDT. Reaction mixtures containing 0.5 μm of the probe and varying concentrations of the protein of interest were prepared in protein‐RNA binding buffer (40 mm Tris‐Cl pH 8, 30 mm KCl, 1 mm MgCl_2_, 0.01% (v/v) Nonidet P40 Substitute (Sigma; 74 385‐1L), 1 mm DTT, 5% (v/v) glycerol, 10 μg·mL^−1^ BSA), adjusted to a final volume of 20 μL. The mixtures were incubated at room temperature for 30 min in the dark.

The native gels for EMSAs (5% polyacrylamide, 0.5× TBE and 2.5% glycerol) were prerun in 0.5x TBE (45 mm Tris, 45 mm boric acid, 1 mm EDTA pH 8) at 100 V for at least 30 min before loading the samples. After the prerun, samples were mixed with Orange G loading dye and run at 100 V until the loading dye reached the bottom of the gel. Imaging was performed using the Odyssey M Imaging System.

### Statistical analysis

Statistical analyses were performed using GraphPad Prism (versions 9 and 10). The specific statistical tests used and their corresponding significance levels are described in the figure legends.

## Results

**Fig. 1:**
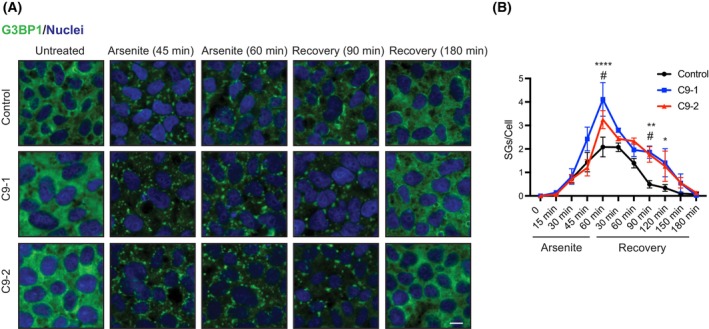
Amyotrophic lateral sclerosis (ALS)‐causing *C9orf72* mutations induce alterations in stress granule (SG) dynamics. (A) Immunocytochemistry in control and C9‐ALS induced pluripotent stem cells (iPSCs) during treatment with 500 μm arsenite and at the indicated time points following arsenite removal (recovery). G3BP1 and Hoechst 33342 staining were used as markers of SGs and nuclei, respectively. Images are representative of three independent experiments. Scale bar: 10 μm. (B) Quantification of total G3BP1‐positive SGs per cell in iPSC cultures (mean ± s.e.m.). Data were obtained from three independent experiments, with at least 200 cells counted per condition. Statistical comparisons were made by two‐way ANOVA with Šidák multiple‐comparison test. *P* values: * C9‐1 vs. control,  *P* < 0.05; **C9‐1 vs. control, *P* < 0.01; ****C9‐1 vs. control, *P* < 0.0001; #C9‐2 vs. control, *P* < 0.05.

Cumulative evidence suggests that SGs act as a nidus for pathological protein aggregation, particularly when they lose their dynamic properties and transition into persistent granules [41, 47, 77, 78]. Moreover, ALS‐causing mutations, including abnormal hexanucleotide expansions in *C9orf72*, induce SG alterations [19, 25, 26, 28, 29, 47, 58, 79, 80, 81, 82, 83]. To gain insight into the pathogenic interplay between SGs and ALS, we monitored SG assembly and disassembly in two patient‐derived iPSC lines carrying distinct *C9orf72*‐HRE mutations (C9‐1: ~6000–8000 bp repeat expansion; C9‐2: ~2400 bp repeat expansion). To this end, we quantified the number of granules per cell containing the SG core protein G3BP1 at various time points during arsenite‐induced oxidative stress and following stress removal (Fig. 1A,B). To confirm that these granules represent SGs, we performed double immunostaining for G3BP1 and TIA1, another well‐established SG marker (Fig. S1A,B). The majority of G3BP1‐positive granules formed upon arsenite treatment were also positive for TIA1 in both control and C9 cells, supporting that these structures are indeed SGs (Fig. S1A,B).

Both C9‐ALS mutant lines (C9‐1 and C9‐2) exhibited an increased number of SGs after 1 h of arsenite treatment compared to controls. Notably, C9‐1 cells, which harbor a longer HRE, displayed elevated SG numbers upon arsenite treatment compared to C9‐2 (Fig. 1A,B). During recovery, both mutant cell lines exhibited slower SG disassembly than controls (Fig. 1A,B). This trend persisted until 3 h post recovery, at which point SG disassembly was complete in both control and mutant *C9orf72*‐expressing lines (Fig. 1A,B).

**Fig. 2:**
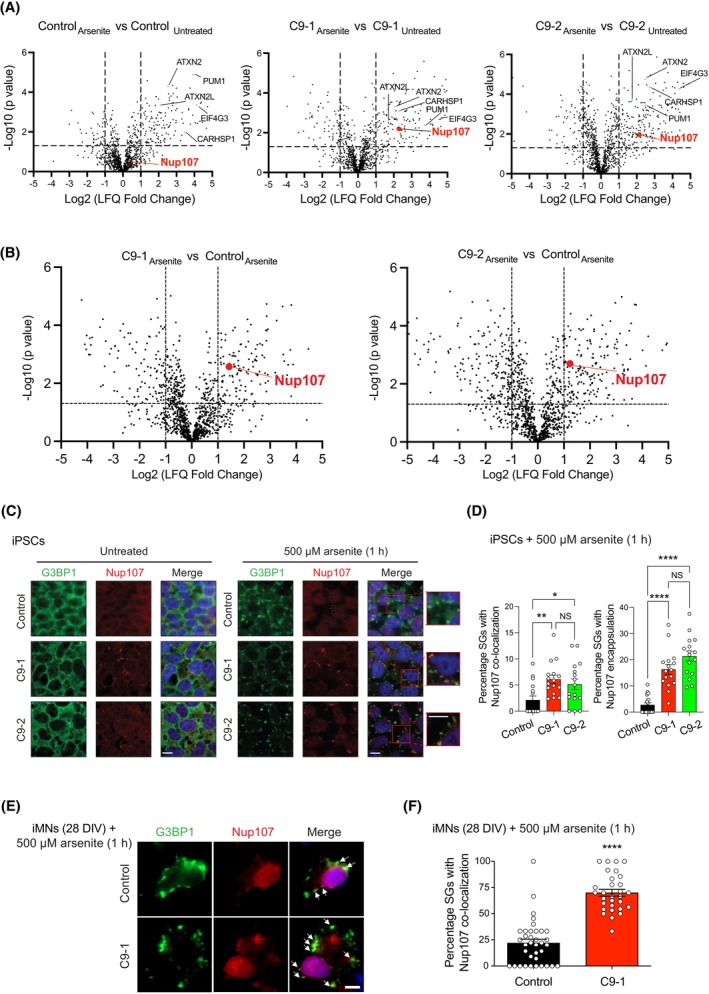
G3BP1 gains enhanced interaction with Nup107 in mutant *C9orf72* motor neurons upon stress granule (SG) assembly. (A) Volcano plots comparing the G3BP1 interactome in control and *C9orf72* motor neurons (C9‐1: ~6000–8000 bp repeat expansion; C9‐2: ~2400 bp repeat expansion), with and without 500 μm sodium arsenite treatment (1 h). The −log_10_ (*P* value) of a two‐sided *t*‐test is plotted against the log_2_‐transformed fold change of protein label‐free quantification (LFQ) values from immunoprecipitation with anti‐G3BP1 antibody (*n* = 3 biological replicates). In green, examples of upregulated interactions with SG core components upon arsenite treatment. (B) Volcano plots of the G3BP1 interactome in *C9orf72* motor neurons compared to control motor neurons treated with 500 μm sodium arsenite for 1 h. (C) Immunocytochemistry using G3BP1 and Nup107 antibodies in control and C9‐ALS induced pluripotent stem cells (iPSCs) under basal conditions (untreated) or after treatment with 500 μm sodium arsenite for 1 h. Hoechst 33342 staining (blue) was used to label nuclei. Images are representative of three independent experiments. Scale bar: 10 μm. (D) Percentage of SGs co‐localizing with or encapsulating Nup107 foci in iPSCs treated with 500 μm sodium arsenite for 1 h (mean ± s.e.m.; *n* = 16 quadrants from 3 independent experiments). Statistical comparisons were made by one‐way ANOVA with Tukey's multiple comparisons test (*P* values: *P < 0.05, **P < 0.01, ****P < 0.0001, NS = Not significant). (E) Immunocytochemistry with G3BP1 and Nup107 antibodies in iPSC‐derived motor neurons (iMNs) treated with 500 μm sodium arsenite (1 h) after 28 days *in vitro* (DIV). Hoechst 33342 staining (blue) was used as a marker of nuclei. Images are representative of two independent experiments. Arrow indicates colocalization of SGs with Nup107 foci. Scale bar: 5 μm. (F) Percentage of SGs co‐localizing with Nup107 foci in iMNs (28 DIV) treated with 500 μm sodium arsenite for 1 h (mean ± s.e.m.; Control *n* = 36 neurons; C9‐1 *n* = 30 neurons from 2 independent experiments). Statistical analysis was made by two‐tailed Student's *t*‐test for unpaired samples (****P < 0.0001).

Given that SG composition influences their dynamics, we analyzed changes in the interactome of G3BP1 in iPSC‐derived motor neurons (iMNs). To this end, we performed co‐immunoprecipitation of G3BP1 followed by label‐free proteomics under both basal and arsenite‐induced SG assembly. As expected, we observed increased interaction of G3BP1 with various SG components following arsenite treatment in both control and C9 motor neurons (Fig. 2A and Table S2). Upon SG assembly, we identified 40 proteins having increased interaction with G3BP1 in both C9‐1 and C9‐2 mutant lines compared to control motor neurons (Fig. 2B and Table S2). Among them, we detected several neurodegeneration‐relevant proteins, such as HSPH1 [84], UBA1 [85], SEPT9 [86], and DDX6 [87] (Fig. 2B and Table S2). We confirmed by immunostaining experiments that DDX6 foci colocalize with G3BP1‐positive granules following arsenite treatment (Fig. S2).

Moreover, the interaction between G3BP1 and the nucleoporin Nup107 was significantly upregulated in both mutant *C9orf72* motor neurons upon SG formation following arsenite treatment, but not in control motor neurons (Fig. 2A and Table S2). Although we also observed increased interactions of G3BP1 with the nucleoporins Nup93 and Nup210 in C9‐1 motor neurons following SG assembly, these interactions were not detected in C9‐2 cells (Table S2). Thus, Nup107 was the only NPC subunit significantly enriched in G3BP1 pulldowns from both mutant *C9orf72‐expressing* motor neurons upon SG induction (Fig. 2A,B and Table S2). We focused on Nup107 due to its critical role in nucleocytoplasmic transport, a pathway widely implicated in C9‐ALS, although the specific contributions of individual components remain unclear [50, 52, 53, 88, 89, 90].

To validate our proteomics findings, we performed immunostaining experiments using antibodies against G3BP1 and Nup107. In undifferentiated control iPSCs, Nup107 exhibited a cytoplasmic and perinuclear distribution under both basal conditions and arsenite treatment (Fig. 2C). In contrast, C9‐ALS iPSCs displayed cytoplasmic Nup107 foci even in the absence of stress (Fig. 2C). Upon arsenite‐induced stress, these Nup107 foci either colocalized with or were encapsulated by SGs (Fig. 2C,D). Following differentiation into motor neurons, iMNs treated with arsenite after two days *in vitro* did not show colocalization of Nup107 with SGs (Fig. S3A). However, when motor neurons were treated with arsenite after 28 days *in vitro*, we observed a significant increase in the percentage of SGs co‐localizing with Nup107 foci in C9‐ALS iMNs compared to controls (Fig. 2E,F). Together, these results suggest that mutant *C9orf72*–induced mislocalization of Nup107 into SGs worsens over time in motor neurons. Unlike undifferentiated iPSCs, iMNs showed only colocalization of Nup107 foci and SGs, but not encapsulation (Fig. 2E). Using an antibody against choline acetyltransferase (ChAT), we confirmed that the cells exhibiting Nup107 and SG colocalization were motor neurons (Fig. S3B).

Nup107 is a component of the Y‐complex, a critical subcomplex required for NPC assembly [91]. To determine whether other structurally essential NPC subunits exhibit altered intracellular distribution in C9‐ALS cells, we screened additional components of the Y‐complex. Besides Nup107, none of the other five tested Y‐complex proteins showed SG colocalization or changes in intracellular distribution in C9‐ALS cells compared to controls (Figs S4 and S5). Additionally, to assess whether Nup107 mislocalization is specific to C9‐ALS, we examined cells expressing ALS‐related FUS^P525L^ and TDP‐43^M337V^ mutant variants. However, mutant FUS and TDP‐43 cells did not exhibit altered Nup107 distribution or colocalization with SGs (Fig. S6).

**Fig. 3:**
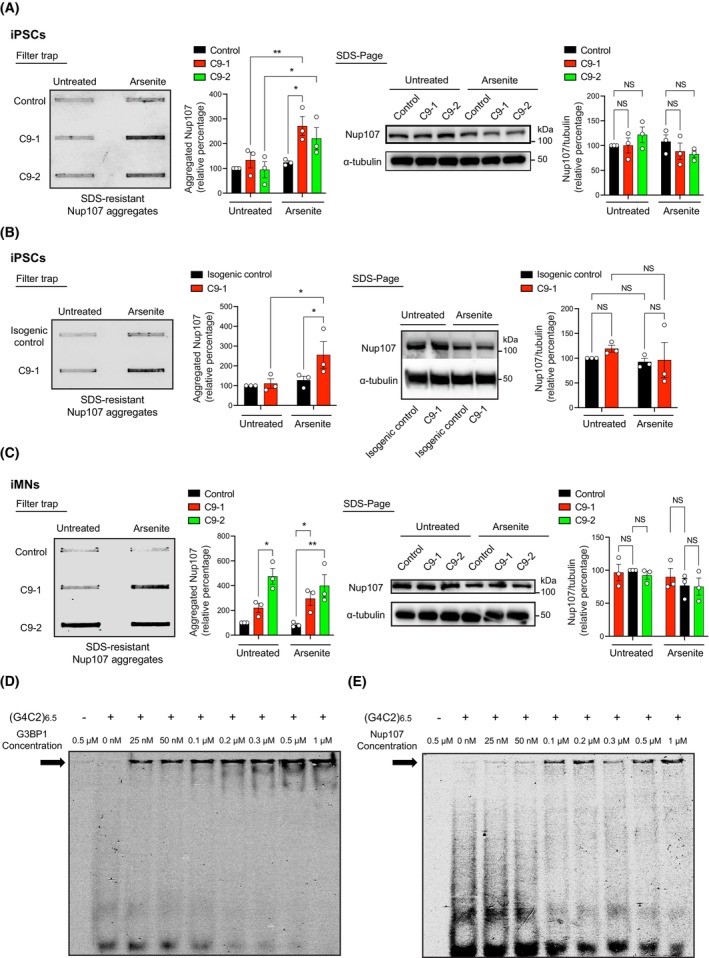
Nup107 aggregates in C9‐ALS cells and binds to pathological *G*_*4*_ *C*_*2*_ RNA repeats. (A) Filter trap assay detecting Nup107 aggregation (anti‐Nup107 antibody) in control and C9‐ALS induced pluripotent stem cells (iPSCs) under basal conditions (untreated) and following treatment with 500 μm arsenite for 1 h. Right: SDS–polyacrylamide gel electrophoresis (SDS/PAGE) with antibodies to Nup107 and α‐tubulin. Graphs represent the relative percentage values of aggregated Nup107 and total Nup107 levels (corrected for α‐tubulin loading control) to untreated control iPSCs (mean ± s.e.m., *n* = 3 independent experiments). Statistical comparisons were made by two‐way ANOVA with Tukey's multiple‐comparison test (*P* values: *P < 0.05, **P < 0.01, NS = Not significant). (B) Filter trap assay detecting Nup107 aggregation in C9‐1 iPSCs and their isogenic controls, in which the *C9orf72* mutation was corrected via CRISPR‐Cas9. Right: SDS/PAGE with antibodies to Nup107 and α‐tubulin. Graphs represent the relative percentage values of aggregated Nup107 and total Nup107 levels (corrected for α‐tubulin) to untreated isogenic control iPSCs (mean ± s.e.m., *n* = 3 independent experiments). Statistical comparisons were made by two‐way ANOVA with Fisher's LSD test (*P* values: * *P* < 0.05, NS = Not significant). (C) Filter trap of Nup107 aggregates in control and C9‐ALS iPSC‐derived motor neurons (iMNs) under basal conditions and after 1 h of 500 μm sodium arsenite treatment. Right: SDS/PAGE with antibodies to Nup107 and α‐tubulin. Graphs represent the relative percentage values of aggregated Nup107 and total Nup107 levels (corrected for α‐tubulin) to untreated control iMNs (mean ± s.e.m., *n* = 3 independent experiments). Statistical comparisons were made by two‐way ANOVA with Tukey's multiple‐comparison test (*P* values: *P < 0.05, **P < 0.01, NS = Not significant). (D, E) Electrophoretic mobility shift assays (EMSA) using purified recombinant G3BP1 (D) or Nup107 (E) titrated with fluorescently labeled (*G4C2*)_6.5_ RNA. The presence of the probe and protein concentrations are indicated at the top. Arrows indicate repeat RNA bound to recombinant protein. Images are representative of two independent experiments.

Protein aggregation is a hallmark of ALS pathology, and certain nucleoporins can aggregate with molecular crowders or during aging, contributing to proteotoxic stress [92, 93, 94]. Although Nup107 lacks the phenylalanine‐glycine repeats typically associated with nucleoporin aggregation, its intrinsically disordered region may promote aggregation (Fig. S7A). To determine whether Nup107 aggregates in C9‐ALS cells, we performed filter trap experiments to detect SDS‐insoluble species. Under normal conditions, we did not observe Nup107 aggregation in undifferentiated C9‐ALS iPSCs (Fig. 3A). However, arsenite treatment led to the accumulation of Nup107 aggregates in these cells (Fig. 3A). In contrast, control iPSCs did not exhibit Nup107 aggregation under the same treatment, despite expressing comparable total Nup107 protein levels (Fig. 3A). We obtained similar results when we compared C9‐1 iPSCs with isogenic controls, in which the *C9orf72* mutation was corrected using CRISPR‐Cas9 (Fig. 3B). To further assess changes in Nup107 aggregation, we separated soluble and insoluble protein fractions followed by western blot analysis. Indeed, we found increased levels of Nup107 in the insoluble fraction of C9 iPSCs following arsenite‐induced SG formation compared to control cells (Fig. S7B). Given that motor neurons are the primary cell type affected in ALS, we next examined iMNs. Notably, C9‐ALS iMNs displayed elevated Nup107 aggregation even in the absence of arsenite treatment (Fig. 3C).

Prompted by these findings, we investigated whether HRE‐derived *G*_*4*_ *C*_*2*_ RNA repeats directly interact with G3BP1 and Nup107, given that RNA‐protein interactions can change protein solubility [95, 96, 97, 98]. To assess binding affinity, we performed electrophoretic mobility shift assays (EMSA) by titrating purified recombinant G3BP1 and Nup107 with fluorescently labeled (*G4C2*)_6.5_ RNA (Fig. 3D,E). G3BP1 bound repeat RNA even at low concentrations (25 nm), as indicated by increased retention of the RNA‐protein complex in the gel well (Fig. 3D). This binding was dose‐dependent, with increasing G3BP1 levels leading to a reduction in free RNA at the bottom of the gel (Fig. 3D). Although Nup107 also bound *G*_*4*_ *C*_*2*_ RNA repeats, it exhibited a weaker binding affinity, with detectable interaction starting at 100 nm (Fig. 3E). In contrast, neither G3BP1 nor Nup107 bound to control (*A*_*4*_ *C*_*2*_)_6.5_, a non‐C9 repeat sequence (Fig. S8A,B). These findings suggest that pathogenic *G*_*4*_ *C*_*2*_ RNA repeats selectively and directly interact with G3BP1 and Nup107, which may contribute to their aggregation.

Given the increased interaction between the SG core component G3BP1 and Nup107, as well as altered SG dynamics in C9‐ALS cells, we asked whether loss of Nup107 can rescue this phenotype. To test this, we knocked down *Nup107* using a doxycycline‐inducible shRNA system (Fig. S9A,B). However, *Nup107* knockdown did not ameliorate the altered SG dynamics observed in C9 cells (Fig. S9C). These results suggest that, although C9 cells display Nup107 aggregation and mislocalization, these changes might not be major drivers of SG dysregulation. We next examined whether *Nup107* knockdown affects cell viability. However, we did not find a reduction in necroptosis and apoptosis markers following *Nup107* knockdown in C9 cells (Fig. S9D).

**Fig. 4:**
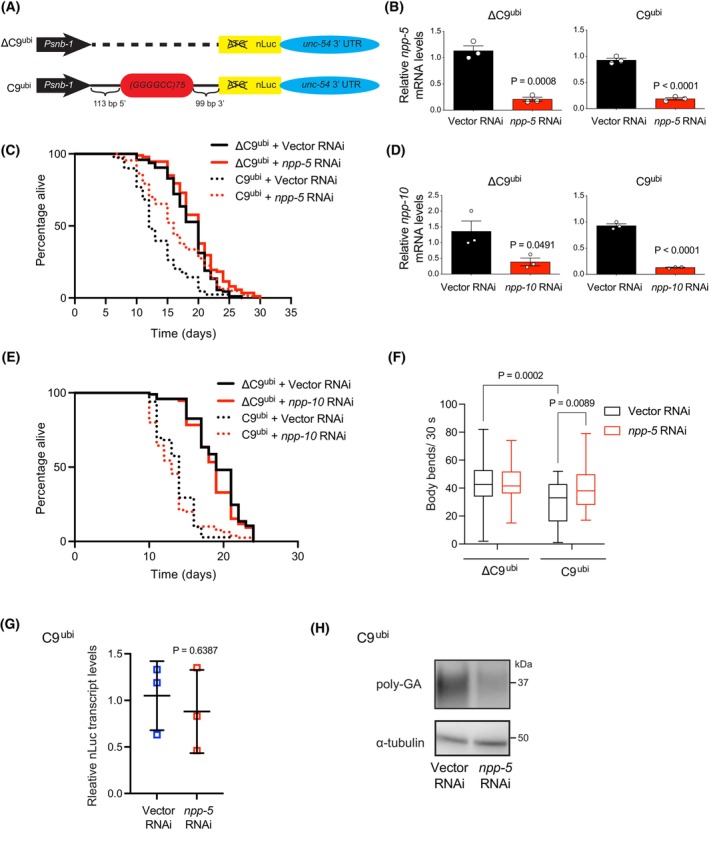
Knockdown of *npp‐5*, the *C. elegans Nup107* ortholog, ameliorates disease‐related phenotypes in C9‐ALS worm models. (A) Schematic representation of transgenic constructs ΔC9^ubi^ and C9^ubi^ for C9‐ALS modeling in *C. elegans*. The schematic is adapted from the original publication describing the generation of these strains. (B) *npp‐5* transcript levels upon RNAi treatment. Graphs represent the relative expression to Vector RNAi control (mean ± s.e.m., *n* = 3 biological replicates). (C) ALS *C9* ^*ubi*^ worms have a shorter lifespan compared to control *ΔC9* ^*ubi*^ worms (*P* < 0.0001). Knockdown of *npp‐5* after development alleviates the short lifespan phenotype of ALS *C9* ^*ubi*^ worms (*P* < 0.0001). *ΔC9* ^*ubi*^ + vector RNAi mean ± s.e.m.: 18.66 days ± 0.37; *ΔC9* ^*ubi*^ + *npp‐5* RNAi: 19.72 ± 0.42; *C9* ^*ubi*^ + vector RNAi: 13.55 ± 0.44; *C9* ^*ubi*^ + *npp‐5* RNAi: 16.48 ± 0.57. *P*‐values: two‐sided log‐rank test, *n* = 96 worms/condition. (D) *npp‐10* transcript levels upon RNAi treatment. Graphs represent the relative expression to Vector RNAi control (mean ± s.e.m., *n* = 3 biological replicates). (E) Knockdown of the nucleoporin *npp‐10/NUP98* after development does not affect the lifespan of control *ΔC9* ^*ubi*^ (*P* = 0.2117) and ALS *C9* ^*ubi*^ worms (*P* = 0.3064). *ΔC9* ^*ubi*^ + vector RNAi mean ± s.e.m.: 19.15 days ± 0.35; *ΔC9* ^*ubi*^ + *npp‐10* RNAi: 18.57 ± 0.33; *C9* ^*ubi*^ + vector RNAi: 13.69 ± 0.28; *C9* ^*ubi*^ + *npp‐10* RNAi: 13.38 ± 0.34. *P*‐values: two‐sided log‐rank test, *n* = 96 worms/condition. (F) Body bends over a 30‐s period in control and C9‐ALS worms at day 5 of adulthood (*n* = 40 worms per condition). Box plots represent the 25th–75th percentiles, the lines depict the median, and the whiskers show the minimum–maximum values. (G) qPCR analysis of *nLuc* transcript levels in ALS *C9* ^*ubi*^ worms at day 5 of adulthood (relative expression to Vector RNAi, mean ± SD, *n* = 3 biological replicates). (H) Western blot analysis of poly‐GA levels in *C9* ^*ubi*^ worms at day 5 of adulthood. α‐tubulin is the loading control. Images are representative of 4 independent experiments. In all experiments, RNAi treatment was initiated after development. Statistical comparisons were made by two‐tailed Student's *t*‐test for unpaired samples (B, D, G) and two‐way ANOVA with Fisher's LSD test (F). Table S3 contains statistical analysis and replicate data from independent lifespan experiments.

Although we did not observe a rescue of SG dynamics or a clear survival benefit *in vitro*, previous work has shown that loss of Nup107 can suppress the rough eye phenotype induced by expanded *G*_*4*_ *C*_*2*_ repeats in Drosophila [50]. To further investigate the potential *in vivo* relevance of Nup107, we next turned to a well‐established *C. elegans* model of C9‐ALS expressing 75 repeats of the *G*_*4*_ *C*_*2*_ HRE under the ubiquitous *snb‐1* promoter (*C9* ^*ubi*^) [70] (Fig. 4A). As a control, we used worms expressing the same construct without HRE (*ΔC9* ^*ubi*^) (Fig. 4A). *C9* ^*ubi*^ worms exhibit key C9‐ALS pathophysiological features, including DPR production via RAN translation, progressive motility decline, and reduced lifespan compared to *ΔC9* ^*ubi*^ worms [70]. To investigate the role of Nup107 in ALS pathology, we knocked down its *C. elegans* ortholog, *npp‐5*, which shares 43% coverage and 41% sequence similarity with human *Nup107*, as determined by BLAST analysis [99]. To achieve this, worms were fed RNAi bacteria expressing either an empty vector control or *npp‐5*‐targeting RNAi after development. Notably, knockdown of *npp‐5* ameliorated the short lifespan phenotype of *C9* ^*ubi*^ ALS worms (Fig. 4B,C and Table S3), suggesting an improvement in disease pathology. In contrast, knockdown of a different nucleoporin, *npp‐10* (*Nup98* ortholog), did not extend the lifespan of *C9* ^*ubi*^ worms (Fig. 4D,E and Table S3). These results suggest that the rescue effect observed upon *npp‐5* knockdown is not due to a general adaptive response to impaired NPC function, but may reflect a more specific role for NPP‐5/Nup107 in C9‐associated toxicity.

Given that motor dysfunction is a hallmark of ALS, we assessed motility in these worms. ALS *C9* ^*ubi*^ worms exhibited a significant decline in motility compared with *ΔC9* ^*ubi*^ controls at Day 5 of adulthood, a phenotype rescued by *npp‐5* knockdown (Fig. 4F). To determine whether this phenotypic improvement was associated with changes in the transcription of the *G*_*4*_ *C*_*2*_ HRE construct or its translation into DPRs, we examined the expression of poly‐GA, one of the most pathological HRE‐derived DPRs. The *C9* ^*ubi*^ construct includes a nanoluciferase (*nLuc*) tag placed in the poly‐GA reading frame (Fig. 4A). While the transcript levels of *nLuc* remained unchanged upon *npp‐5* knockdown (Fig. 4G), western blot analysis revealed a marked reduction in poly‐GA peptide levels (Fig. 4H). These findings suggest that lowering NPP‐5/Nup107 levels does not affect HRE transcription but instead decreases DPR accumulation from HRE‐derived transcripts.

## Discussion

Dysregulation of SG dynamics and nucleocytoplasmic transport (NCT) is emerging as a hallmark of C9‐ALS; however, the precise links between these processes remain unclear [29, 50, 56, 82, 89]. Our study identifies the nucleoporin Nup107 as a potential factor bridging these two disease‐related changes. We demonstrate that Nup107 is widely mislocalized, associates with SGs, and aggregates in C9‐ALS cells. Notably, our experiments reveal that Nup107 aggregation occurs independently of its total protein levels, suggesting that its accumulation into insoluble aggregates results from misfolding or sequestration rather than protein overexpression.

Besides Nup107, we did not observe other nucleoporins having arsenite‐induced interaction with G3BP1 in the two independent C9 iPSC‐derived cell lines used in our study. However, since not all nucleoporins were detected in our proteomics experiments, we cannot exclude the possibility that other nuclear NPC subunits also interact with G3BP1 following SG assembly. Nup107 is a component of the NPC Y‐complex. We examined five additional nucleoporins from this subcomplex, but none showed accumulation in SGs. One intriguing possibility is that Nup107 accumulation arises from specific interactions with protein or RNA components of SGs. It is important to note that other nucleoporins (e.g., Nup50, Nup62, Nup88, Nup205, and Nup358) have previously been reported to accumulate in SGs in HEK293 cells under certain stress conditions [56]. Nup62, another nucleoporin containing intrinsically disordered regions, has been shown to colocalize with G3BP1 and TDP‐43 in the cytoplasm, contributing to their insolubility [57].

The impact of HRE‐derived RNA on nucleoporin dysfunction remains largely unexplored. We found that Nup107 directly binds *G*_*4*_ *C*_*2*_ repeat RNA, albeit with lower affinity than G3BP1. This suggests that HRE RNA may contribute to Nup107 misfolding or dysregulated intracellular distribution, potentially linking RNA toxicity to SG alterations. Along these lines, previous studies have demonstrated that G3BP1 colocalizes with HRE sense‐ and antisense probes [48, 100]. Furthermore, Zfp106, another HRE‐interacting protein, has been reported to bind Nup107 [75], reinforcing the notion that HRE RNA contributes to nucleoporin dysfunction in C9‐ALS. Given the elevated interaction of Nup107 with G3BP1 and its localization to SGs in C9 ALS, along with the interaction of both Nup107 and G3BP1 with *G*_*4*_ *C*_*2*_ repeat RNA, an intriguing possibility is that HRE *C9orf72*‐derived RNA repeats accumulate within Nup107 foci and SGs in C9 ALS cells. In future studies, fluorescent *in situ* hybridization (FISH) experiments could shed light on this possibility.

To further explore the links between Nup107 and ALS, we examined whether reducing its levels mitigates C9‐ALS phenotypes using *C. elegans* models. Knockdown of *npp‐5*, the *C. elegans* ortholog of *Nup107*, significantly extended lifespan and rescued motor deficits in C9‐ALS worms. These beneficial effects occurred without changes in mutant transcript levels. Instead, we observed a marked reduction in poly‐GA peptide levels, suggesting that Nup107 may influence RAN‐mediated translation into DPRs. Alternatively, Nup107 could influence DPR clearance, reducing its accumulation. Due to the lack of available antibodies against *C. elegans* NPP‐5, we were unable to assess its subcellular localization or potential colocalization with GTBP‐1 (the G3BP1 ortholog) in the C9^ubi^ ALS worm model.

In summary, our results highlight Nup107 as a potential therapeutic target in C9‐ALS, linking RNA toxicity, nucleoporin dysfunction, SG alterations, and protein aggregation. Indeed, our *in vivo* results suggest that reducing NPP‐5/Nup107 levels mitigates ALS‐related changes in worm models. Together, our findings further support a link between nucleoporins and C9‐ALS pathology.

## Conflict of interest

The authors declare no conflict of interest.

## Author contributions

SB and DV conceived the study. SB designed and performed most of the experiments and data analysis. YN and WHZ contributed to motor neuron differentiation and SG analysis. DP contributed to EMSA and *C. elegans* experiments. MW performed protein expression and purification. SMG contributed to lifespan assays. SK performed protein aggregation assays and contributed to additional experiments. SB and DV wrote the manuscript. All authors revised and approved the manuscript.

## Supporting information


**Fig. S1.** The majority of G3BP1‐positive granules formed upon arsenite treatment are also positive for TIA1 in both control and C9 cells.
**Fig. S2.** A fraction of DDX6 foci co‐localize with G3BP1‐positive granules following arsenite treatment.
**Fig. S3.** Nup107 foci do not colocalize with stress granules in early‐stage iPSC‐derived motor neurons (iMNs) but show colocalization at later stages.
**Fig. S4.** ALS‐causing *C9orf72* mutations do not induce colocalization of Nup43 and Nup96/98 outer ring nucleoporins with stress granules.
**Fig. S5.** ALS‐causing *C9orf72* mutations do not induce colocalization of Nup133, Nup160 and Sec13 outer ring nucleoporins with stress granules.
**Fig. S6.** ALS‐causing FUS and TDP‐43 mutations do not induce NUP107 localization within stress granules.
**Fig. S7.** Nup107 levels are elevated in the insoluble protein fraction of C9‐ALS induced pluripotent stem cells (iPSCs) following arsenite‐induced stress granule formation.
**Fig. S8.** Both G3BP1 and Nup107 bind pathogenic *G4C2* RNA repeats but not control *A4C2* RNA repeats.
**Fig. S9.** Knockdown of *Nup107* in C9‐ALS induced pluripotent stem cells (iPSCs) does not rescue alterations in stress granule (SG) dynamics.

**Table S1.** List of primers used in this study.

**Table S2.** Protein interactome of G3BP1 in motor neurons.

**Table S3.** Statistical analysis and replicate data of lifespan experiments.

## Data Availability

The authors declare that the main data supporting the findings of this study are available within the article and its Supporting Information. The proteomics data have been deposited to the ProteomeXchange Consortium (https://proteomecentral.proteomexchange.org) via the PRIDE partner repository (accession code: PXD065424). All the data are also available from the corresponding author upon request.
