# Supplementary material for: C9orf72 ALS‐causing mutations lead to mislocalization and aggregation of nucleoporin Nup107 into stress granules
Source: FEBS Lett. 2025 Sep 1;599(21):3047–65. doi: 10.1002/1873-3468.70156 (PMC12599624; doi:10.1002/1873-3468.70156)
Supplement: Supplementary file 1 — Fig. S1. The majority of G3BP1‐positive granules formed upon arsenite treatment are also positive for TIA1 in both control and C9 cells. Fig. S2. A fraction of DDX6 foci co‐localize with G3BP1‐positive granules following arsenite treatment. Fig. S3. Nup107 foci do not colocalize with stress granules in early‐stage iPSC‐derived motor neurons (iMNs) but show colocalization at later stages. Fig. S4. ALS‐causing C9orf72 mutations do not induce colocalization of Nup43 and Nup96/98 outer ring nucleoporins with stress granules. Fig. S5. ALS‐causing C9orf72 mutations do not induce colocalization of Nup133, Nup160 and Sec13 outer ring nucleoporins with stress granules. Fig. S6. ALS‐causing FUS and TDP‐43 mutations do not induce NUP107 localization within stress granules. Fig. S7. Nup107 levels are elevated in the insoluble protein fraction of C9‐ALS induced pluripotent stem cells (iPSCs) following arsenite‐induced stress granule formation. Fig. S8. Both G3BP1 and Nup107 bind pathogenic G4C2 RNA repeats but not control A4C2 RNA repeats. Fig. S9. Knockdown of Nup107 in C9‐ALS induced pluripotent stem cells (iPSCs) does not rescue alterations in stress granule (SG) dynamics. [file FEB2-599-3047-s004.pdf]

Supplementary Figures

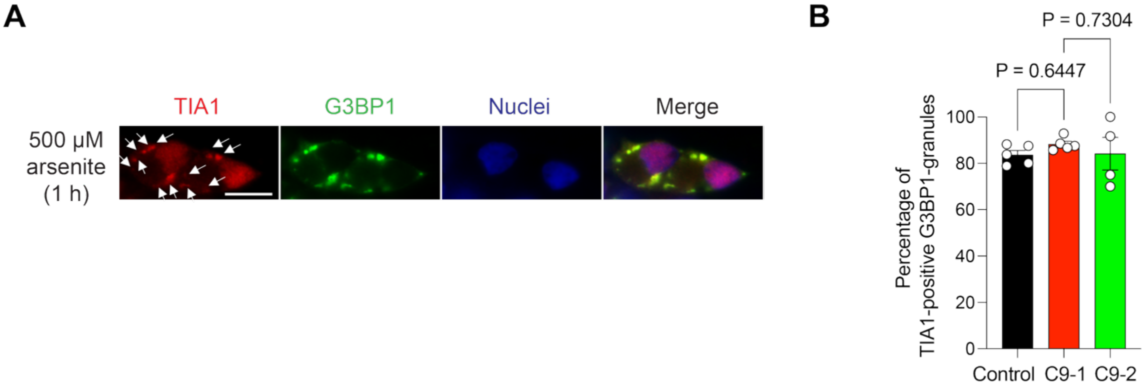

Supplementary Figure S1. The majority of G3BP1-positive granules formed upon arsenite treatment are also positive for TIA1 in both control and C9 cells. (A) Immunocytochemistry with TIA1 and G3BP1 in C9-2 iMNs treated with 500 µM sodium arsenite (1 h). Hoechst 33342 staining (blue) was used as a marker of nuclei. Arrows indicate TIA1 foci co-localizing with G3BP1 foci. Images are representative of two independent experiments. Scale bar: 10 µm. (**B**) Graph represents the percentage of TIA1-positive G3BP1-containing granules in control and C9 cells treated with 500 µM sodium arsenite for 1 h (mean ± s.e.m., Control: n = 5, C9-1: n = 5, C9-1: n = 4 with a total of 15-38 cells counted per condition). Statistical comparisons were made by one-way ANOVA with Tukey's multiple comparisons test.

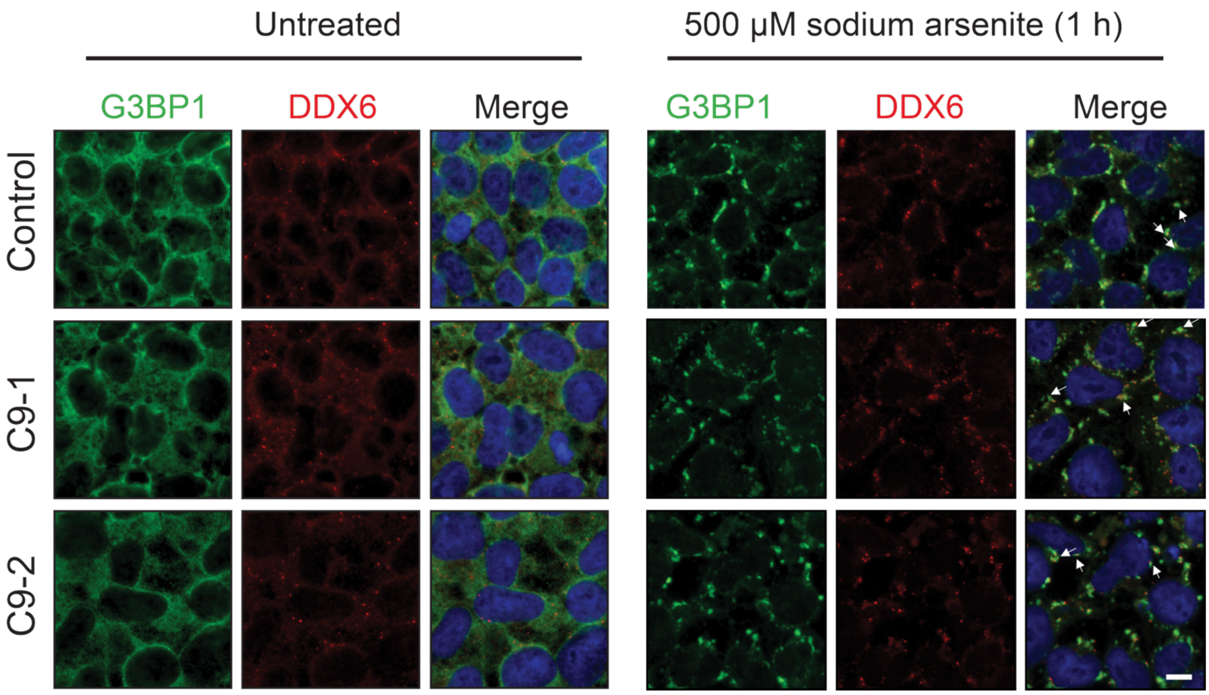

Supplementary Figure S2. A fraction of DDX6 foci co-localize with G3BP1-positive granules following arsenite treatment. Immunostaining of G3BP1 and DDX6 in control and C9-ALS induced pluripotent stem cells (iPSCs) under basal conditions (untreated) or after treatment with 500 µM sodium arsenite for 1 hour. Hoechst 33342 staining was used to label nuclei. Images are representative of two independent experiments. Arrows indicate examples of co-localization between DDX6 and G3BP1 foci. Scale bars: 10 µm.

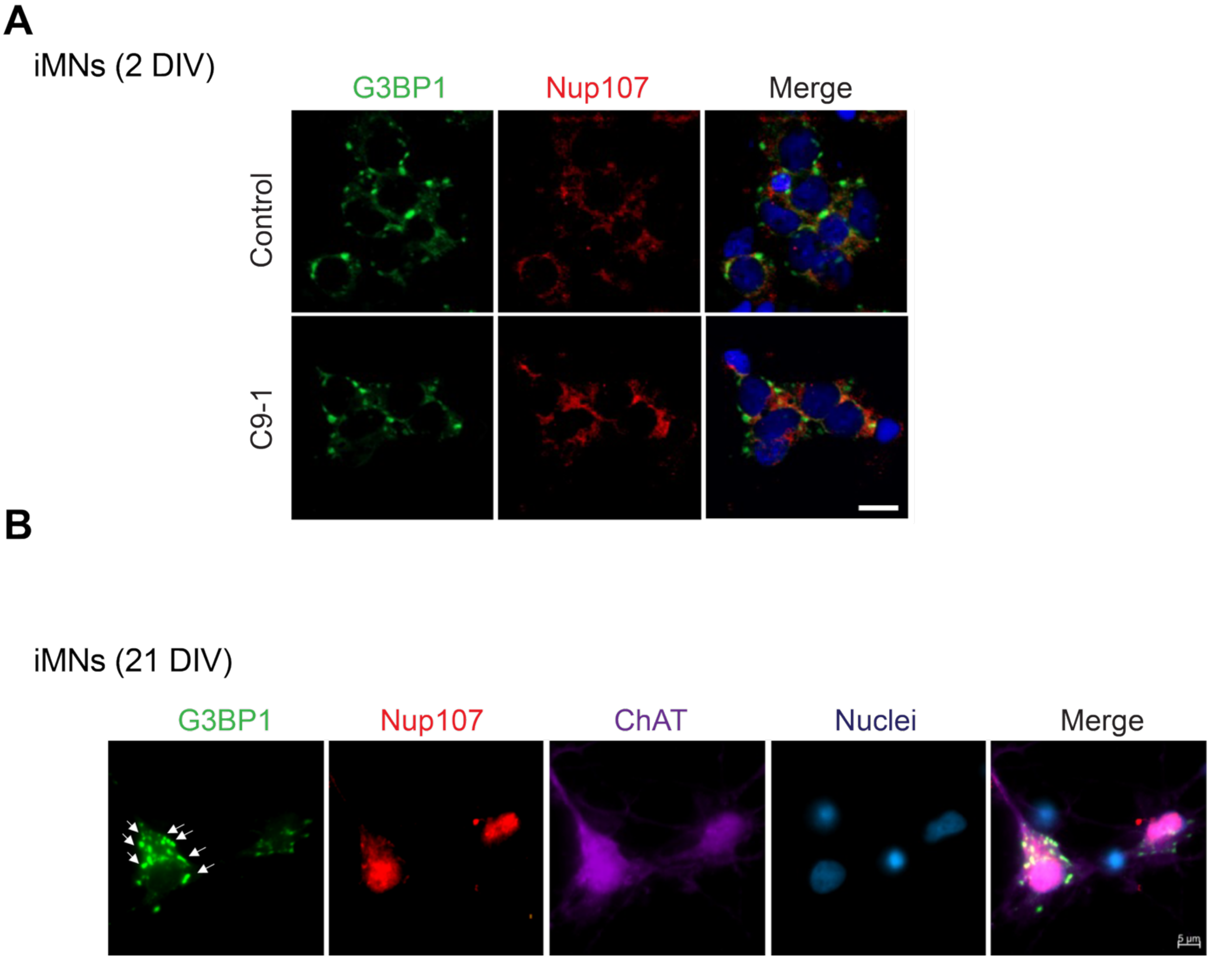

Supplementary Figure S3. Nup107 foci do not co-localize with stress granules in early-stage iPSC-derived motor neurons (iMNs) but show co-localization at later stages. (**A**) Immunocytochemistry with G3BP1 and Nup107 antibodies in iMNs treated with 500 µM sodium arsenite (1 h) after 2 days in vitro (DIV). Hoechst 33342 staining (blue) was used as a marker of nuclei. Images are representative of two independent experiments. Scale bar: 10 µm. (**B**) Immunocytochemistry with G3BP1 and Nup107 antibodies in iMNs treated with 500 µM sodium arsenite (1 h) after 21 DIV. Anti-choline acetyltransferase (ChAT) antibody and Hoechst 33342 staining were used as markers of motor neurons and nuclei, respectively. Images are representative of two independent experiments. Arrows indicate co-localization between NUP107 and G3BP1 foci. Scale bar: 5µm.

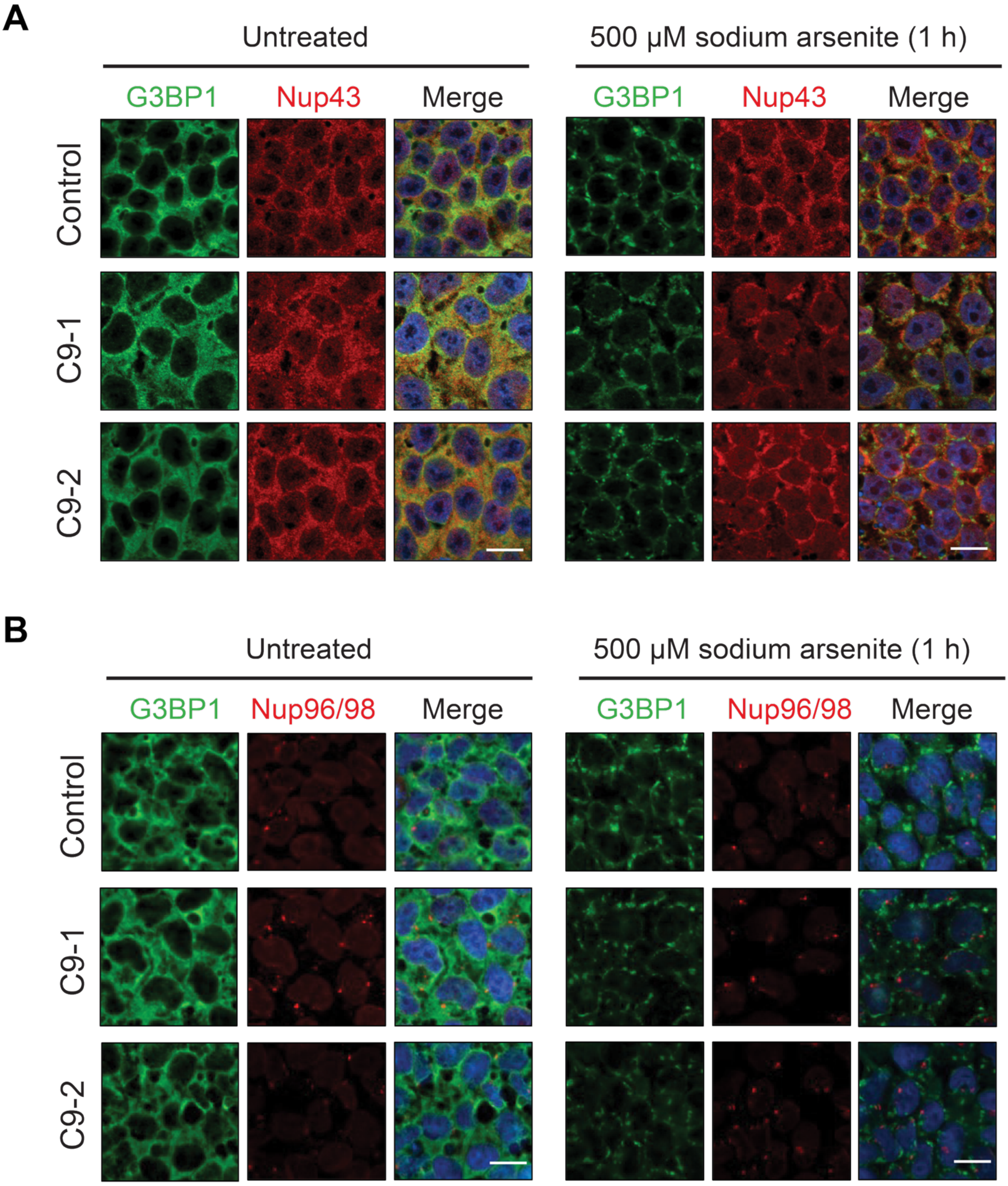

Supplementary Figure S4. ALS-causing *C9orf72* mutations do not induce co-localization of Nup43 and Nup96/98 outer ring nucleoporins with stress granules. Immunostaining of G3BP1 and the Y-complex nucleoporins Nup43 (**A**) and Nup96/98 (**B**) in control and C9-ALS induced pluripotent stem cells (iPSCs) under basal conditions (untreated) or after treatment with 500 μM sodium arsenite for 1 hour. Hoechst 33342 staining was used to label nuclei. Images are representative of three independent experiments. Scale bars: 20 μm.

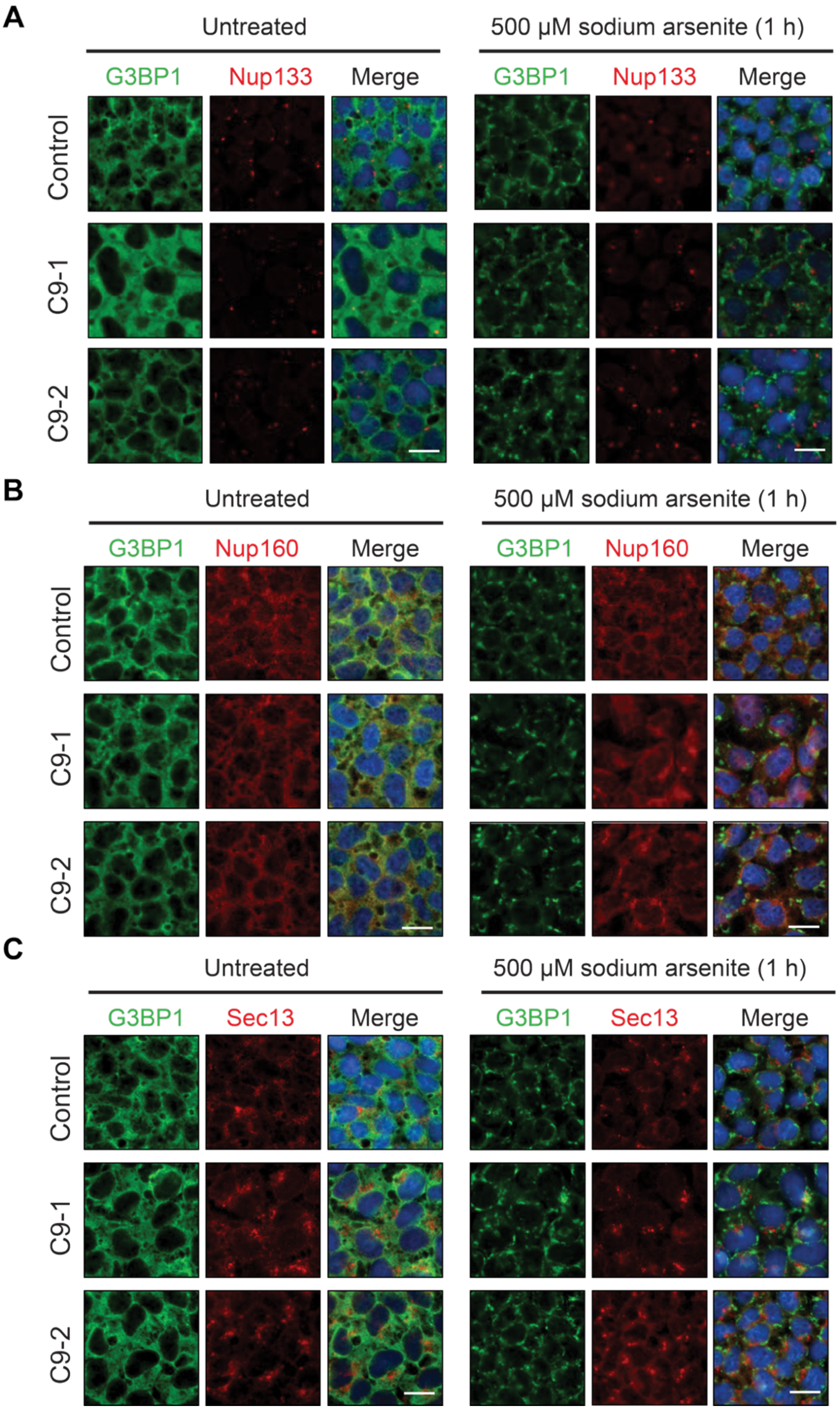

Supplementary Figure S5. ALS-causing *C9orf72* mutations do not induce co-localization of Nup133, Nup160 and Sec13 outer ring nucleoporins with stress granules. Immunostaining of G3BP1 and the Y-complex nucleoporins Nup133 (**A**), Nup160 (**B**) and Sec13 (**C**) in control and C9-ALS induced pluripotent stem cells (iPSCs) under basal conditions (untreated) or after treatment with 500 µM sodium arsenite for 1 hour. Hoechst 33342 staining was used to label nuclei. Images are representative of three independent experiments. Scale bars: 20 µm.

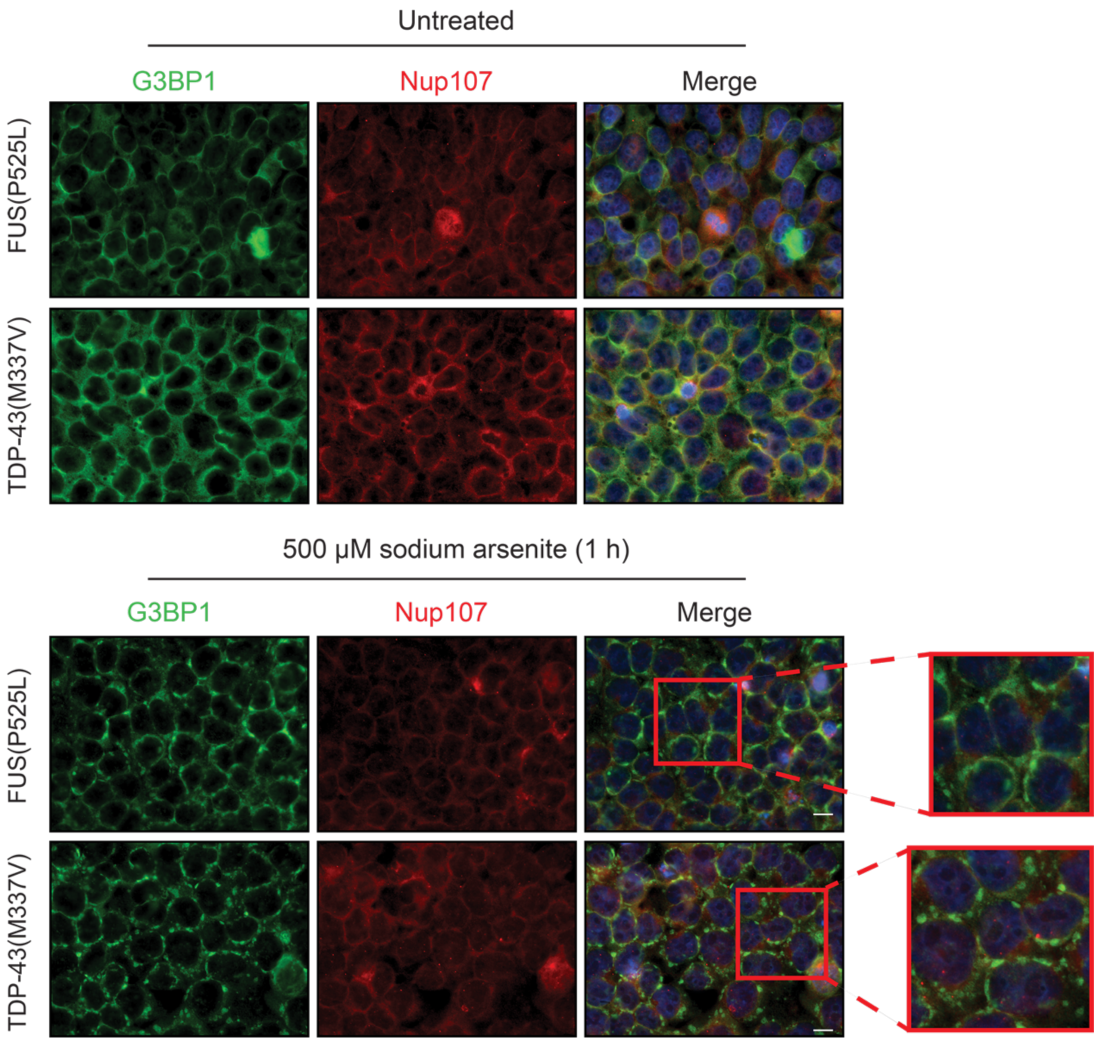

Supplementary Figure S6. ALS-causing FUS and TDP-43 mutations do not induce NUP107 localization within stress granules. Immunostaining of induced pluripotent stem cells (iPSCs) expressing ALS-related mutant FUS and TDP-43 variants after treatment with 500 µM sodium arsenite for 1 hour. Hoechst 33342 staining was used to label nuclei. Images are representative of three independent experiments. Scale bars: 10 µm.

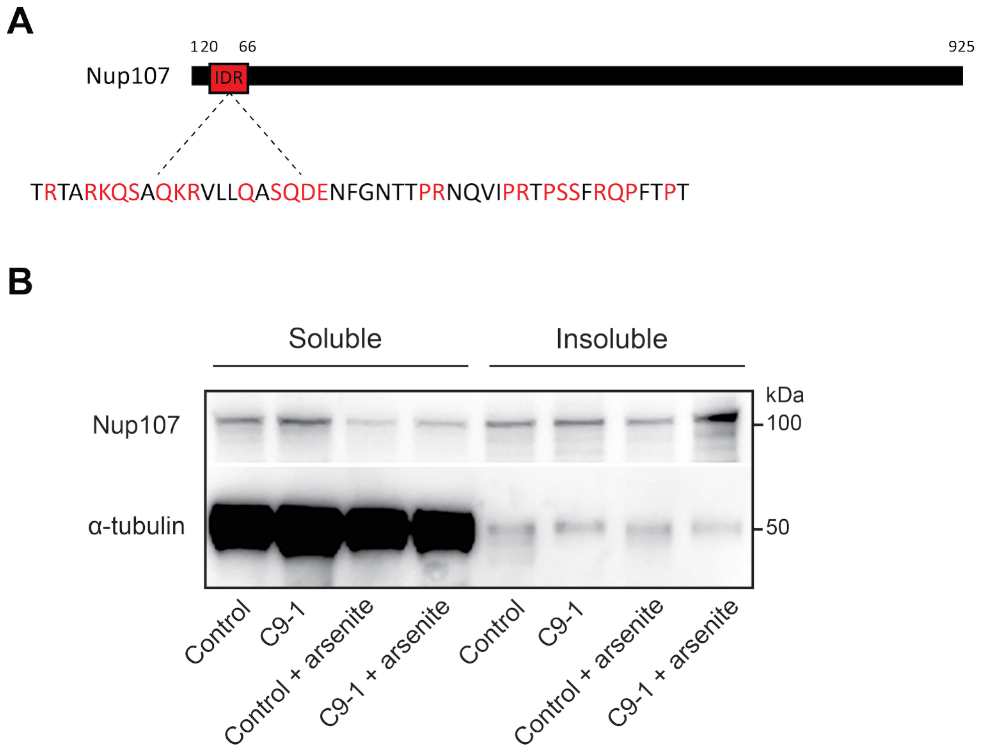

Supplementary Figure S7. Nup107 levels are elevated in the insoluble protein fraction of C9-ALS induced pluripotent stem cells (iPSCs) following arsenite-induced stress granule formation. (**A**) Schematic representation of the intrinsically disordered region of the Nup107 protein. (**B**) Western blot analysis of soluble and insoluble protein fractions from control and C9-ALS iPSCs. Cells were treated with 500 μM arsenite for 1 hour. Images are representative of three independent experiments.

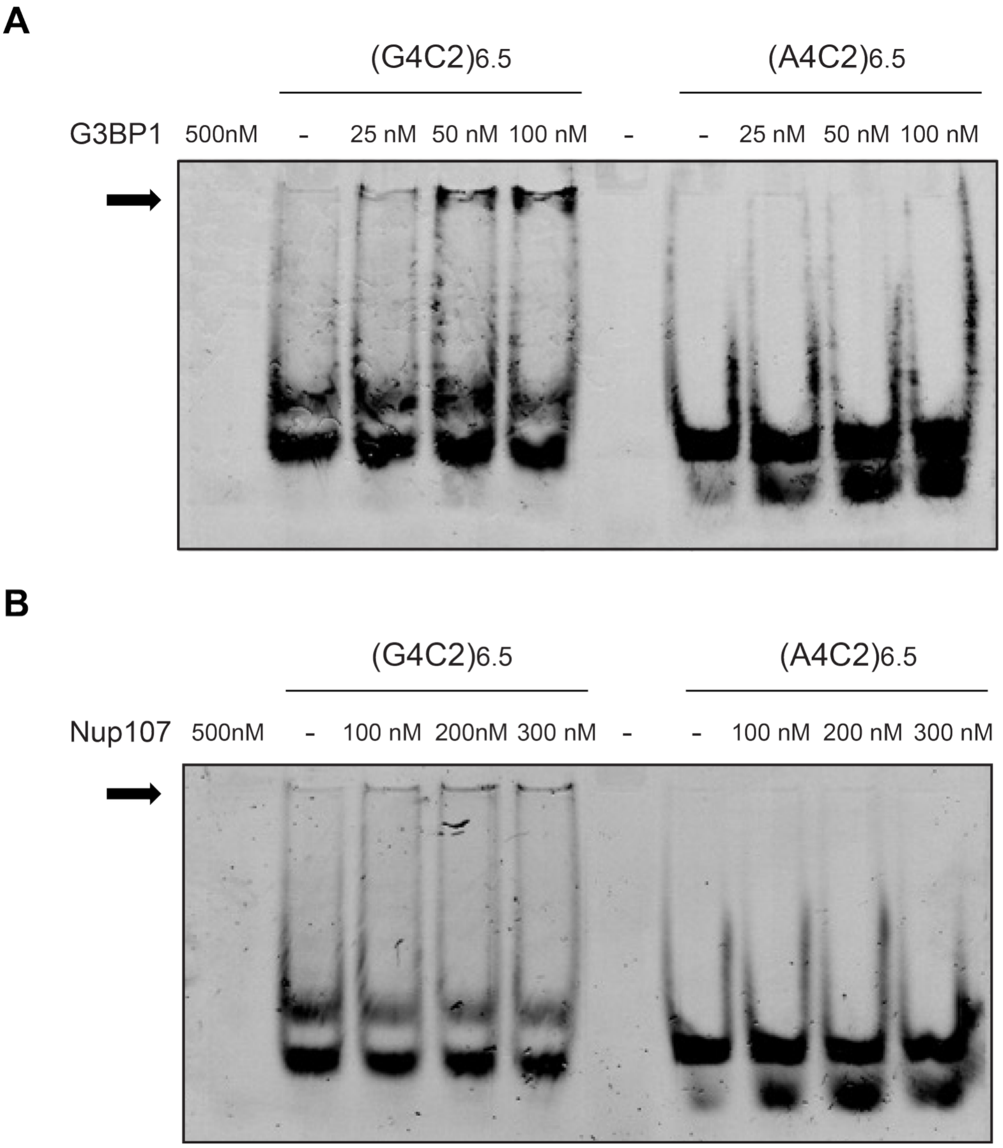

Supplementary Figure S8. Both G3BP1 and Nup107 bind pathogenic *G4C2* RNA repeats but not control *A4C2* RNA repeats. Electrophoretic mobility shift assays (EMSA) using purified recombinant G3BP1 (**A**) or Nup107 (**B**) titrated with fluorescently labeled pathogenic $(\mathit{G4C2})_{6.5}$ or control $(\mathit{A4C2})_{6.5}$ RNA probes. The presence of the probe and protein concentrations are indicated at the top. Arrows indicate repeat *G4C2* RNA repeat bound to recombinant protein. Images are representative of two independent experiments.

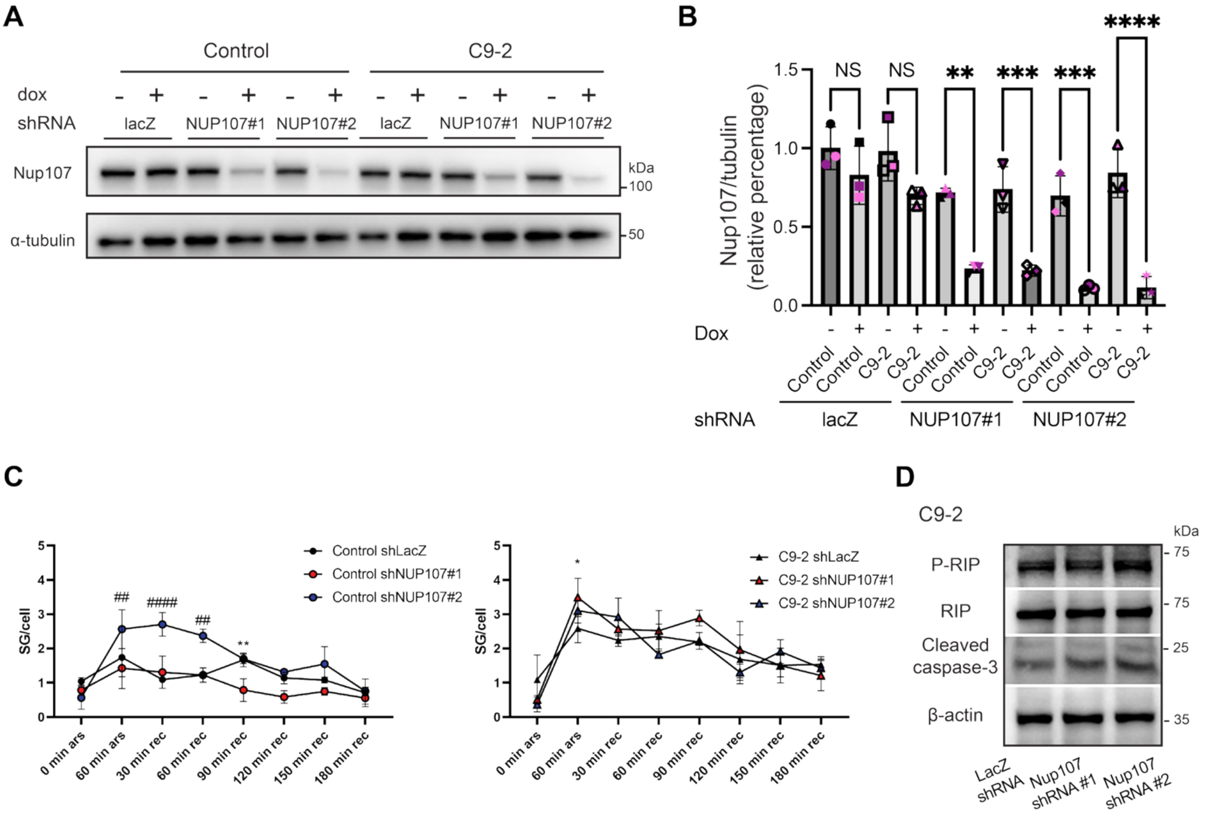

Supplementary Figure S9. Knockdown of *Nup107* in C9-ALS induced pluripotent stem cells (iPSCs) does not rescue alterations in stress granule (SG) dynamics. (**A**) Western blot analysis of Nup107 knockdown levels in control and C9-2 G3BP1::GFP iPSCs. α-tubulin is the loading control. shRNA expression was induced with 2 μg ml^{-1} doxycycline (Dox) treatment for 2 days. Images are representative of three independent experiments. (**B**) Graph represents the relative values of Nup107 protein levels (corrected for α-tubulin loading control) to control iPSCs + lacZ shRNA without Dox treatment (mean ± S.D., $n = 3$ independent experiments). Statistical comparisons were made by one-way ANOVA with Tukey's multiple-comparison test (*P* values: ** $P < 0.01$, *** $P < 0.001$, **** $P < 0.0001$, NS= Not significant). (**C**) Quantification of G3BP1::GFP-positive SGs per cell in control (left) and C9-2 (right) iPSCs expressing lacZ or Nup107 shRNA (mean ± S.D.). shRNA expression was induced with 2 μg ml^{-1} Dox for 2 days. Data were obtained from three independent experiments, with at least 120 cells counted per condition. Statistical comparisons were made by two-way ANOVA with Dunett's multiple-comparison test. *P* values: * shLacZ vs shNup107 #1, $P < 0.05$; ** shLacZ vs shNup107 #1, $P < 0.01$; [##] shLacZ vs shNup107 #2, $P < 0.01$; [####] shLacZ vs shNup107 #2, $P < 0.0001$. (**D**) Western blot analysis of C9 iPSCs expressing control lacZ or Nup107 shRNA with antibodies to phosphorylated RIP at Ser166 (P-RIP), total RIP, cleaved caspase-3 and β-actin loading control. P-RIP and cleaved caspase-3 are markers of necroptosis and apoptosis, respectively. shRNA expression was induced with 2 μg ml^{-1} Dox for 2 days. Images are representative of two independent experiments.
